# Delaying Broccoli Floret Yellowing by Phytosulfokine α Application During Cold Storage

**DOI:** 10.3389/fnut.2021.609217

**Published:** 2021-04-01

**Authors:** Morteza Soleimani Aghdam, Majid Alikhani-Koupaei, Raheleh Khademian

**Affiliations:** ^1^Department of Horticultural Science, Imam Khomeini International University, Qazvin, Iran; ^2^Department of Production Engineering and Plant Genetics, Faculty of Agriculture, Higher Education Complex of Saravan, Saravan, Iran; ^3^Department of Genetic and Plant Breeding, Faculty of Agriculture and Natural Resources, Imam Khomeini International University, Qazvin, Iran

**Keywords:** chlorophyll degradation, ethylene biosynthesis, floret yellowing, hydrogen sulfide, pheophorbide a oxygenase, phytosulfokine α

## Abstract

During postharvest life, broccoli suffers from floret yellowing confining its economic and nutritional value. The objective of the present study was to explore the mechanisms employed by phytosulfokine α (PSKα) at 150 nM for delaying floret yellowing in broccoli during storage at 4°C for 28 days. Our results showed that the higher endogenous accumulation of hydrogen sulfide (H_2_S) resulting from the higher gene expression and activities of l-cysteine desulfhydrase (*LCD*) and d-cysteine desulfhydrase (*DCD*) in broccoli floret treated with 150 nM PSKα may serve as an endogenous signaling molecule for delaying senescence. Moreover, the suppressed ethylene biosynthesis in broccoli floret treated with 150 nM PSKα might be ascribed to lower gene expression and activities of ACC synthase (*ACS*) and ACC oxidase (*ACO*). Furthermore, lower gene expression and activities of Mg^2+^ dechelatase (*MDC*), pheophytinase (*PPH*), and pheophorbide a oxygenase (*PaO*) might be the reasons for the higher accumulation of chlorophyll in broccoli floret treated with 150 nM PSKα. Based on our findings, exogenous PSKα application could be employed as signaling bioactive hormone for retarding floret yellowing of broccoli during storage at 4°C for 28 days.

## Introduction

Owing to higher health-promoting bioactive molecules accumulation, broccoli has gained worldwide attention as a global healthy food crop, which is beneficial for ensuring human health by reducing the risk of chronic diseases, such as cancer, cardiovascular diseases, and neurodegenerative diseases in industrial countries (1).

By harvesting broccoli prematurely, a high accumulation of intracellular reactive oxygen species (ROS) (2), an insufficient intracellular supply of ATP (3, 4), an imbalance of intracellular hormones, signified by a higher biosynthesis of ethylene and a lower biosynthesis of cytokinin (5, 6), and chlorophyll degradation *via* the pheophorbide a oxygenase (PaO) pathway (2, 6–9) may be the mechanisms for the accelerated broccoli senescence, manifested by floret yellowing, which confines its commercial value. In recent years, postharvest procedures, such as 1-methylcyclopropene (1-MCP) (10), nitric oxide (2), brassinolide (6), putrescine (8), hydrogen sulfide (H_2_S) (4, 11), cytokinins (12), folic acid (13), melatonin (14), arginine, cysteine, and methionine (15), and phytosulfokine α (PSKα) (16–18), have been employed by researchers for delaying broccoli senescence manifested by floret yellowing during cold storage.

In plants, providing sufficient cysteine is crucial for H_2_S biosynthesis by l-cysteine desulfhydrase (LCD) enzyme activity in the cytosol and mitochondria and d-cysteine desulfhydrase (DCD) enzyme activity in the mitochondria (19–21). By exogenous H_2_S application, endogenous H_2_S accumulation resulting from higher *LCD* and *DCD* gene expressions and enzyme activities has been beneficial for improving the marketability in horticultural crops by sufficient intracellular ATP supply by promoting H^+^-ATPase, Ca^2+^-ATPase, cytochrome c oxidase (CCO), and succinate dehydrogenase (SDH) enzyme activities, preventing chlorophyll degradation by suppressing chlorophyll b reductase (*CBR*), chlorophyllase (*Chlase*), Mg^2+^ dechelatase (*MDC*), pheophytinase (*PPH*), and *PaO* gene expressions and enzyme activities, promoting endogenous proline accumulation by triggering pyrroline-5-carboxylate synthase (*P5CS*) and ornithine aminotransferase (*OAT*) gene expressions and enzyme activities along with hindering proline dehydrogenase (*PDH*) gene expression and enzyme activity, promoting ROS scavenging superoxide dismutase (*SOD*), catalase (*CAT*), ascorbate peroxidase (*APX*), and glutathione reductase (*GR*) gene expressions and enzyme activities leading to lower ${\text{O}}_{2}^{-}$ and H_2_O_2_ accumulation, keeping membrane integrity by presenting lower electrolyte leakage and malondialdehyde (MDA) accumulation resulting from lower phospholipase D (PLD) and lipoxygenase (LOX) enzyme activities, preventing ethylene biosynthesis by suppressing ACC synthase (*ACS*) and ACC oxidase (*ACO*) gene expressions and enzyme activities, promoting phenylpropanoid pathway activity by presenting higher phenylalanine ammonia lyase (PAL)/polyphenol oxidase (PPO) enzyme activity leading to higher phenols, flavonoids, and anthocyanins accumulation, promoting oxidative pentose phosphate pathway activity by presenting higher glucose 6-phosphate dehydrogenase (G6PDH) and 6-phosphogluconate dehydrogenase (6PGDH) enzyme activities for supplying NADPH and erythritol 4-phosphate, and promoting chitinase and β-1,3-glucanase gene expressions and enzyme activities (4, 22–30).

PSKα [Tyr(SO_3_H)-Ile-Tyr(SO_3_H)-Thr-Gln] is a bioactive signaling tyrosine-disulfate pentapeptide hormone biosynthesized from a prepropeptide of 80–120 amino acid prepropeptides encoded by the *PSKs* gene (31, 32). By promoting cytosolic second messenger guanosine 3′,5′-cyclic monophosphate (cGMP) accumulation by employing exogenous PSKα application resulting from phytosulfokine receptor 1 (PAR1) kinase encapsulating guanylate cyclase activity, triggering cytosolic Ca^2+^ accumulation may be responsible for triggering friendly extracellular ATP and intracellular SnRK1 signaling pathways, triggering SUMO E3 ligase SIZ1 system activity accompanied by suppressing NAD^+^ dissipating poly(ADP-ribose) polymerase 1 (PARP1) system activity for ensuring sufficient intracellular ATP supply resulting from higher H^+^-ATPase, Ca^2+^-ATPase, CCO, and SDH enzyme activities accompanied by sufficient intracellular NADPH supply resulting from higher G6PDH, 6PGDH, and methylenetetrahydrofolate dehydrogenase (MTHFD) enzyme activities, triggering ROS avoiding alternative oxidase (*AOX*) and uncoupling protein (*UCP1*) gene expressions and scavenging *SOD, CAT, APX*, and *GR* gene expressions and enzyme activities, promoting phenylpropanoid pathway activity by presenting higher *PAL* and chalcone synthase (*CHS*) gene expressions and enzyme activities leading to higher phenols, flavonoids, and anthocyanins accumulation, promoting ascorbic acid accumulation resulting from higher l-galactono-1,4-lactone dehydrogenase (*GLDH*) gene expression along with lower ascorbic acid oxidase (*AAO*) gene expression, promoting endogenous melatonin accumulation resulting from higher tryptophan decarboxylase (*TDC*), tryptamine 5-hydroxylase (*T5H*), serotonin N-acetyltransferase (*SNAT*), and N-acetylserotonin O-methyltransferase (*ASMT*) gene expressions, promoting endogenous cytokinin accumulation resulting from higher isopentenyl transferase (*IPT*) gene expression concomitant with lower cytokinin oxidase (*CKO*) gene expression, suppressing *PLD* and *LOX* gene expressions and enzyme activities, triggering heat shock protein (*HSP70* and *HSP90*) gene expressions, and improving 2,2-diphenyl-1-picrylhydrazyl (DPPH), ferric ion reducing antioxidant power (FRAP), and DPPH scavenging capacity resulting from higher phenols, flavonoids, anthocyanins, and ascorbic acid accumulation may be responsible for attenuating chilling injury and fungal decay, delaying senescence and maintaining the nutritional quality of fruits and vegetables during cold storage (16–18, 33–37). However, there are ongoing attempts to introduce safe and operative procedures for avoiding floret yellowing and preserving the floret quality of broccoli during cold storage.

Therefore, triggering endogenous PSKα signaling pathway by exogenous PSKα application or endogenous PSKα accumulation may be efficient for relieving chilling injury and fungal decay, delaying senescence and keeping the quality of horticultural crops during postharvest life (16–18, 33–37). The present study aimed to investigate the connection between exogenous PSKα application and broccoli floret yellowing and elucidate its mechanisms from the perspective of endogenous H_2_S accumulation by *LCD* and *DCD* gene expressions and enzyme activities, chlorophyll degradation by *MDC, PPH*, and *PaO* gene expressions, and enzyme activities accompanied by ethylene biosynthesis by *ACS* and *ACO* gene expressions and enzyme activities by employing exogenous PSKα application at 0 and 150 nM during storage at 4°C for 28 days.

## Materials and Methods

### Broccoli and PSKα Application

Broccoli (*Brassica oleracea* var. italica) was harvested at commercial maturity when the individual floret was still closed and was dark green and the inflorescence was compact. Healthy broccoli heads with uniform size, color, and maturity stage were selected for PSKα treatment. PSKα (soluble in sterile water, 1 mg/ml) was provided by Pepmic Co., Ltd., Suzhou, China. For PSKα treatment, 240 broccoli heads were allocated into two groups of 120 (40 heads per replicate) for the experimental treatments, applied by spraying the heads with 25 ml of PSKα at 0 (control; double distilled water, ddH_2_O) or 150 nM according to Aghdam et al. (16) and Aghdam and Luo (18). After drying overnight, the broccoli heads were packaged in polyethylene bags (50 ×80 cm, 0.04 mm) and stored at 4 ± 0.5°C and 85% relative humidity for 28 days. Then, every 7 days during storage at 4°C, floret from 10 broccolis was excised, immediately frozen in liquid nitrogen, powdered, and stored at −80°C for biochemical and gene expression analysis. For biochemical and gene expression analysis, three technical replications were carried out by three extractions from each biological replication to avoid instrumental error. The means of three technical replications are considered as one biological replication.

### Broccoli Floret Yellowing

Broccoli floret yellowing (%) was assessed by a scale from 0 to 9 as stated by Shi et al. (2), where 0 indicates all dark green, 1 indicates 25% yellowing, 5 indicates 50% yellowing, 7 indicates 75% yellowing, and 9 indicates 100% yellowing of the broccoli floret.

### Chlorophyll Degradation by MDC and PPH Enzyme Activities

Chlorophyll accumulation in broccoli floret was measured according to Gómez-Lobato et al. (38), by homogenizing 1 g of frozen powder with 10 ml of 80% (v/v) acetone and centrifugation at 10,000 × *g* for 10 min at 4°C. By measuring absorbance at 663 and 645 nm, chlorophyll accumulation was calculated using the formula:

Chlorophyll accumulation (g kg^−1^ FW) = (17.76 × A_645nm_) + (7.34 × A_663nm_).

For analyzing MDC and PPH enzyme activities, 1 g of frozen powder was homogenized with 10 ml of cold acetone. After centrifugation at 12,000 × *g* for 5 min at 4°C, the precipitate was used for assaying MDC and PPH enzyme activities according to 38. MDC enzyme activity was assayed by pheophorbide a formation at 686 nm and was expressed in mkatals produced per mass of protein, mkat kg^−1^. PPH enzyme activity was assayed by pheophorbide a formation at 667 nm and was expressed in mkatals produced per mass of protein, mkat kg^−1^.

### Ethylene Biosynthesis by ACS and ACO Enzyme Activities

Ethylene production was measured using gas chromatography as stated by Fan et al. (39). Ethylene production was expressed as μg kg^−1^ h^−1^ on a fresh weight basis. For ACS enzyme activity, 1 g of frozen powder was homogenized with 5 ml of 100 mM sodium phosphate buffer (pH 9.0) containing 5 μM pyridoxal phosphate, 4 mM β-mercaptoethanol, 1 mM ethylenediaminetetraacetic acid (EDTA), and 10% (v/v) glycerol. After centrifugation at 16,000 × *g* for 20 min at 4°C, the supernatant was used for ACS enzyme activity assaying according to Suzuki et al. (40). ACS activity was expressed based on ACC production as μmol kg^−1^ h^−1^ on a fresh weight basis. For ACO enzyme activity, 1 g of frozen powder was homogenized with 10 ml of 100 mM Tris–HCl buffer (pH 7.2) containing 30% (w/v) glycerol, 10 mM sodium ascorbate, and 5 mM dithiothreitol (DTT). After centrifugation at 14,000 × *g* for 20 min at 4°C, the supernatant was used for ACO enzyme activity assaying according to Suzuki et al. (41). ACO activity was expressed based on ethylene production as μmol kg^−1^ h^−1^ on a fresh weight basis.

### Endogenous H_2_S Accumulation by LCD and DCD Enzyme Activities

For endogenous H_2_S accumulation assaying by methylene blue method as stated by Li et al. (4), 1 g of frozen powder was homogenized with 10 ml of 50 mM phosphate buffer saline (pH 6.8) containing 0.2 M ascorbic acid and 0.1 M EDTA. After centrifugation at 10,000 × *g* for 20 min, the supernatants were mixed in a test tube containing 0.1 M phosphate buffer saline (pH 7.4), 2 mM phosphopyridoxal, and 10 mM l-cysteine. The released H_2_S was absorbed in a zinc acetate trap. The absorbance was measured at 667 nm, and the calibration curve was established with Na_2_S solution concentrations. Endogenous H_2_S accumulation was expressed as μmol kg^−1^ on a fresh weight basis. The LCD and DCD enzyme activities were analyzed as stated by Li et al. (4). Then, 1 g of frozen powder was homogenized with 20 mM Tris–HCl (pH 8.0). The homogenate was centrifuged at 12,000 × *g* for 20 min. For LCD and DCD enzyme activity assays, 1 ml of supernatant was mixed with 1 ml of mixture solution containing 100 mM Tris–HCl (pH 9.0 for LCD enzyme and 8.0 for DCD enzyme), 0.8 mM l-cysteine for LCD enzyme and d-cysteine for DCD enzyme, and 2.5 mM DTT. After incubation at 37°C for 15 min, the reaction was terminated by adding 100 μl of 30 mM FeCl_3_ dissolved in 1.2 M HCl and 100 μl of 20 mM N,N-dimethyl-p-phenylenediamine (DMPD) dissolved in 7.2 M HCl. Then, the formation of methylene blue was recorded at 670 nm. A calibration curve was established with Na_2_S solution concentrations. The LCD and DCD enzyme activities were expressed on a fresh weight basis as μmol kg^−1^ h^−1^.

### Genes Expression Assay by RT-qPCR

A 1 g of frozen powder was used for total RNA extraction from broccoli floret by Trizol reagent (Invitrogen, CA, USA). To confirm total RNA quantity and quality, we determined the absorbance at 260 nm by using a Thermo Scientific™ NanoDrop™ One Spectrophotometer and 1.0% agarose gel electrophoresis. The cDNA was synthesized from 2 μg of total RNA using the SuperScript® RT (Invitrogen, CA, USA) kit according to the manufacturer's instructions. The cDNA was used as a template for assaying the relative expression of genes by quantitative reverse transcription-PCR (RT-qPCR) utilizing a StepOne™ Real-Time PCR System. The final volume of 10 μl containing 1 μl of cDNA, 100 nM primers (Supplementary Table 1), and 5 μl of 2 × SYBR GREEN I Master Mix (TaKaRa, Japan) was prepared according to the instruction provided by the manufacturer. All primers' specificity was determined using the BLAST sequence alignment with Primer-BLAST software. To better quantify the relative expression of the genes, the threshold cycle (Ct) value was normalized to the *Actin* Ct value and calculated following 2^−Δ*ΔCt*^ according to Livak and Schmittgen (42).

### Statistical Analysis

The experiment was planned using split plots in a time model based on a completely randomized design (CRD). For biochemical and genes expression analysis, three technical replications were carried out by three extractions from each biological replication to avoid instrumental error. The means of three technical replications are considered as one biological replication. All data were expressed as mean ± standard error (SE) from three biological replications. ANOVA was carried out, and the mean was compared using Tukey's test at a significance level of 0.01 using the SPSS software (version 19.0).

## Results and Discussion

### Broccoli Floret Yellowing and Ethylene Production

**Figure 1:**
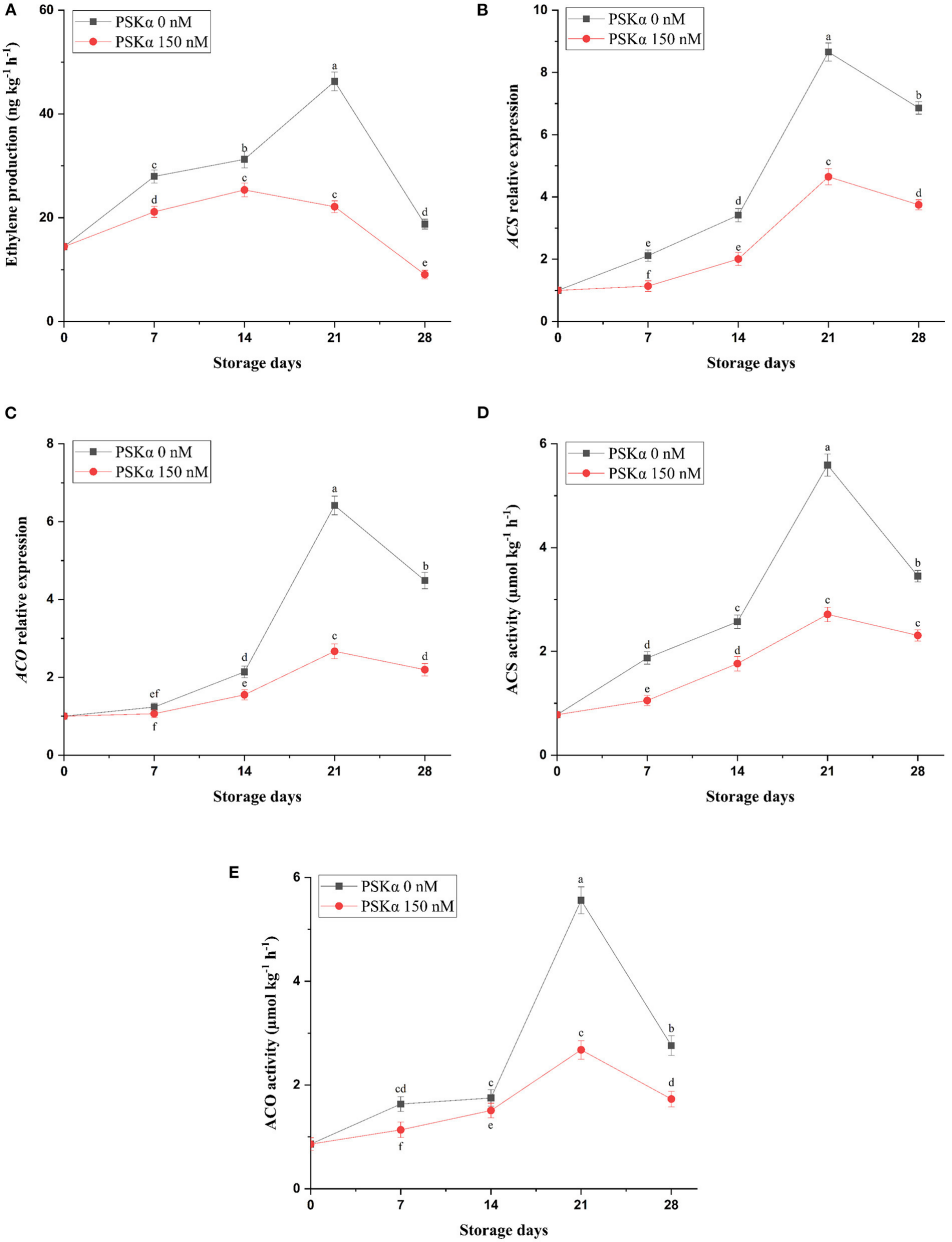
Ethylene production **(A)** accompanied by *ACS* and *ACO* gene expressions **(B,C)** and enzyme activities **(D,E)** in broccoli floret treated with PSKα at 0 and 150 nM during storage at 4°C for 28 days. Data shown are mean values of *n* = 3, and the error bars represent standard errors of the means. Tukey's test at *P* = 0.05 level.

As depicted in Supplementary Figure 1, broccoli treated with PSKα at 150 nM exhibited the lowest floret yellowing during storage at 4°C for 28 days (*P* < 0.01). Additionally, retarding floret yellowing in broccoli treated with PSKα at 150 nM was concomitant with lower ethylene production (*P* < 0.01; Figure 1), which might be attributed to lower *ACS1* and *ACO1* gene expressions and enzyme activities (*P* < 0.01; Figure 1) during storage at 4°C for 28 days. During postharvest life, higher ethylene biosynthesis by *ACS* and *ACO* gene expressions and enzyme activities may be the reason for triggering floret yellowing in broccoli by promoting membrane phospholipids degradation by *PLD* gene expression and enzyme activity leading to free fatty acids supplying for peroxidation by *LOX* gene expression and enzyme activity. Higher LOX activity not only is liable for deteriorating membrane fluidity and integrity signifying higher MDA accumulation but also is liable for endogenous jasmonic acid accumulation for accelerating broccoli floret yellowing by motivating *CBR, PPH*, and *PaO* gene expressions and enzyme activities (38, 43, 44). Hence, suppressing ethylene biosynthesis accompanied by hampering membrane deteriorating LOX enzymes activity by exogenous 1-MCP and exogenous cytokinin application or *IPT* gene overexpression has been employed successfully for suppressing floret yellowing in broccoli during postharvest life (5, 40, 45, 46). According to our results, lower ethylene production in broccoli floret treated with PSKα at 150 nM might be attributed to lower *ACS1* and *ACO1* gene expressions and enzyme activities during storage at 4°C for 28 days. Hence, low temperature storage (4°C) synergistically by exogenous PSKα application may be crucial for suppressing ethylene production efficient for suppressing floret senescence manifested by yellowing.

### Broccoli Floret Chlorophyll Degradation

**Figure 2:**
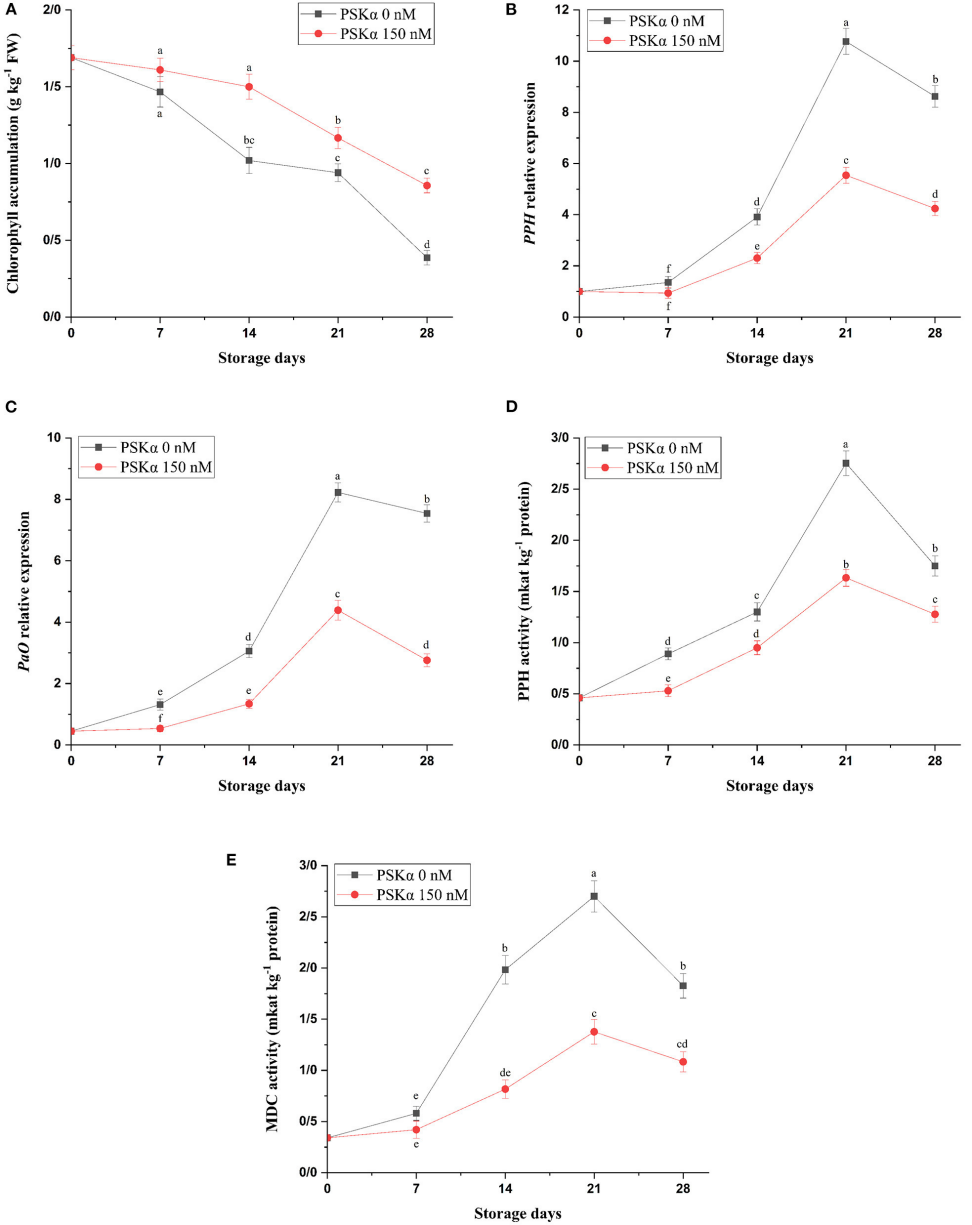
Chlorophyll accumulation **(A)** accompanied by *PPH* and *PAO* gene expressions **(B,C)** and MDC and PPH enzyme activities **(D,E)** in broccoli floret treated with PSKα at 0 and 150 nM during storage at 4°C for 28 days. Data shown are mean values of *n* = 3, and the error bars represent standard errors of the means. Tukey's test at *P* = 0.05 level.

As depicted in Figure 2, broccoli treated with PSKα at 150 nM exhibited higher chlorophyll accumulation (P < 0.01; Figure 2), which might be attributed to lower *PPH* and *PaO* gene expressions (*P* < 0.01; Figure 2) accompanied by lower MDC and PPH enzyme activities (*P* < 0.01; Figure 2) during storage at 4°C for 28 days. During postharvest senescence, PaO pathway activity is liable for chlorophyll degradation. During PAO pathway activity, Chlase enzyme activity is liable for phytol removal from chlorophyll a leading to chlorophyllide a producing in the thylakoid membrane. Chen et al. (47) reported that the silencing *Chlase* gene expression suppressed floret yellowing during postharvest life. After chlorophyll a dephytylation by Chlase, MDC enzyme activity is liable for Mg^2+^ removing from chlorophyllide a producing pheophorbide a in chloroplast stroma (48). In broccoli floret during senescence, demetallation may be the reason for producing pheophytin a from chlorophyll a by MDC enzyme activity. Then, PPH enzyme activity is liable for producing pheophorbide a in chloroplast stroma from pheophytin a (49–51). Suppressing floret yellowing in broccoli by cytokinin treatment might be ascribed to repressing *PPH* gene expression, and accelerating floret yellowing in broccoli by ethylene treatment might be ascribed to enhancing *PPH* gene expression (50). Then, PaO enzyme activity is liable for producing red chlorophyll catabolite (RCC) from pheophorbide a by oxidative chlorine ring-opening (48). Cai et al. (6) reported that suppressing floret yellowing in broccoli by exogenous brassinolide application might be ascribed to lower ethylene biosynthesis resulting from lower *ACS* and *ACO* gene expressions accompanied by higher chlorophyll accumulation resulting from lower *Chlase, PPH*, and *PaO* gene expressions. Hence, higher chlorophyll accumulation in broccoli floret treated with PSKα at 150 nM might be attributed to lower *PPH* and *PaO* gene expressions accompanied by lower MDC and PPH enzyme activities during storage at 4°C for 28 days.

### Broccoli Floret Endogenous H_2_S Accumulation

**Figure 3:**
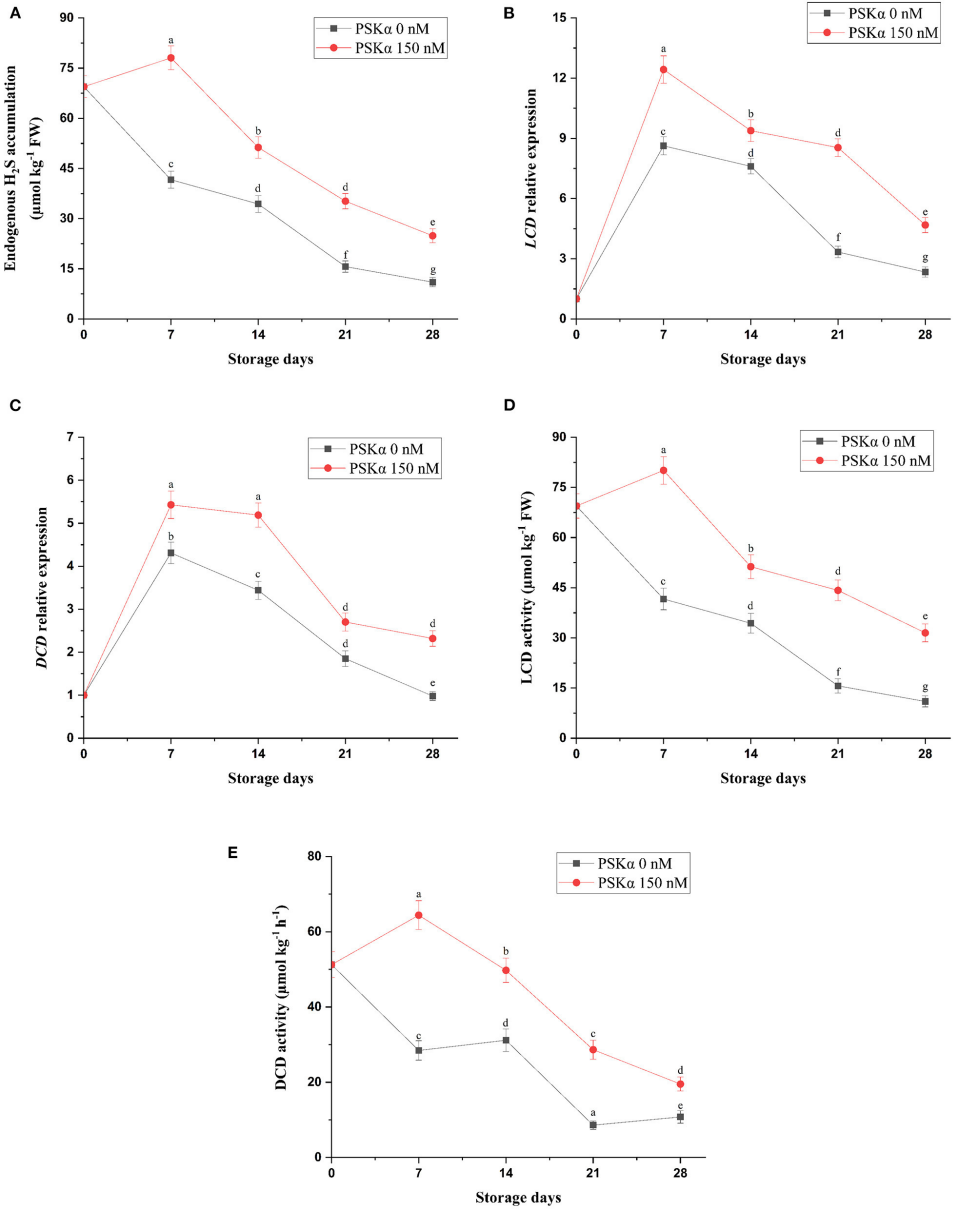
Endogenous H_2_S accumulation **(A)** accompanied by *LCD* and *DCD* gene expressions **(B,C)** and enzyme activities **(D,E)** in broccoli floret treated with PSKα at 0 and 150 nM during storage at 4°C for 28 days. Data shown are mean values of *n* = 3, and the error bars represent standard errors of the means. Tukey's test at *P* = 0.05 level.

As depicted in Figure 3, broccoli treated with PSKα at 150 nM exhibited higher endogenous H_2_S accumulation (*P* < 0.01; Figure 3), which might be attributed to higher *LCD* and *DCD* gene expressions and enzyme activities (*P* < 0.01; Figure 3) during storage at 4°C for 28 days. Hu et al. (22) reported that suppressing leaf yellowing in spinach treated with H_2_S might be ascribed to endogenous H_2_S accumulation resulting from higher LCD and DCD enzyme activities efficient for sufficient intracellular ATP supply resulting from electron transferring from sulfide to CCO for accelerating electron transport system activity accompanied by higher chlorophyll accumulation resulting from lower Chlase and MDC enzyme activities along with higher SOD and CAT enzyme activities leading to higher DPPH and ${\text{O}}_{2}^{-}$ scavenging capacity, keeping membrane integrity signifying lower electrolyte leakage and MDA accumulation. Li et al. (4) reported that suppressing floret yellowing in broccoli treated with H_2_S might be ascribed to endogenous H_2_S accumulation resulting from higher LCD and DCD enzyme activities efficient for sufficient intracellular ATP supply resulting from higher H^+^-ATPase, Ca^2+^-ATPase, SDH, and CCO enzyme activities accompanied by sufficient intracellular NADPH supply resulting from higher G6PDH and 6PGDH enzyme activities. Liu et al. (26) reported that suppressing daylily flower senescence treated with H_2_S might be ascribed to endogenous H_2_S accumulation resulting from higher LCD and DCD enzyme activities efficient for sufficient intracellular ATP supply resulting from higher H^+^-ATPase, Ca^2+^-ATPase, SDH, and CCO enzyme activities accompanied by higher SOD, CAT, and APX enzyme activities leading to lower ${\text{O}}_{2}^{-}$ and H_2_O_2_ accumulation, keeping membrane integrity signifying lower MDA accumulation. Aghdam et al. (27) reported that relieving chilling damage in hawthorn fruit treated with H_2_S might be ascribed to endogenous H_2_S accumulation resulting from higher LCD and DCD enzyme activities efficient for higher SOD, CAT, and APX enzyme activities leading to lower H_2_O_2_ accumulation; higher phenols, flavonoids, and anthocyanins accumulation; and higher DPPH scavenging activity resulting from higher PAL enzyme activity, keeping membrane integrity signifying lower MDA accumulation. Therefore, higher endogenous H_2_S accumulation in broccoli floret treated with PSKα at 150 nM might be attributed to higher *LCD* and *DCD* gene expressions and enzyme activities during storage at 4°C for 28 days efficient for suppressing floret yellowing by suppressing ethylene biosynthesis and chlorophyll degradation.

Endogenous H_2_S accumulation serves as a signaling molecule by persulfidation, a protein post-translational modification (PTM). By exogenous H_2_S application or endogenous H_2_S accumulation, NADPH oxidase, LCD, APX, and glyceraldehyde 3-phosphate dehydrogenase (GAPDH) activity upregulation and ACO, NADP-isocitrate dehydrogenase (NADP-ICDH), NADP-malic enzyme (NADP-ME), and CAT activity downregulation have been attributed to persulfidation (52). González-Gordo et al. (53) suggested that the endogenous H_2_S accumulation regulates the metabolism of plant organelles cytosol, chloroplast, mitochondrion, and peroxisome by persulfidation. Jia et al. (28) reported that the endogenous accumulation of H_2_S suppresses ethylene biosynthesis by inhibiting the activity of ACO following cysteine persulfidation. Recently, Li et al. (54) reported that the H_2_S conferred oxidative stress tolerance to tomato plants by persulfidation of ROS scavenging CAT and APX. Therefore, possibly, the endogenous accumulation of H_2_S suppresses ethylene biosynthesis, due to lower activities of ACS and ACO and a suppressed chlorophyll degradation due to lower activities of MDC and PPH by protein persulfidation. However, this assumption needs to be validated in future studies by employing other methods, such as an improved tag-switch method for the *in situ* labeling of intracellular persulfides (55).

## Conclusion

To sum up, our results demonstrated that the exogenous PSKα application at 150 nM may be employed as a promising procedure for delaying senescence of broccoli floret by promoting endogenous H_2_S accumulation resulting from higher *LCD* and *DCD* gene expressions and enzyme activities giving to suppressing ethylene production resulting from lower *ACS1* and *ACO1* gene expressions and enzyme activities accompanied by higher chlorophyll accumulation resulting from lower *PPH* and *PaO* gene expressions concomitant with lower MDC and PPH enzyme activities during storage at 4°C for 28 days. The present study demonstrated the efficiency of 150 nM PSKα treatment as a preservation procedure for postharvest broccoli to delay floret yellowing, and thus the use of 150 nM PSKα may have broad application prospects. Thus, exogenous PSKα application can be employed as a beneficial strategy for delaying the senescence of broccoli floret during low temperature storage.

## Data Availability Statement

The raw data supporting the conclusions of this article will be made available by the authors, without undue reservation.

## Author Contributions

MSA conceived the idea and wrote the manuscript. MSA, MA-K, and RK performed the experiments and supported the data analysis. All the authors read, discussed, and approved the manuscript.

## Conflict of Interest

The authors declare that the research was conducted in the absence of any commercial or financial relationships that could be construed as a potential conflict of interest.
