# Supplementary figures and images for: Delaying Broccoli Floret Yellowing by Phytosulfokine α Application During Cold Storage
Source: Front Nutr. 2021 Apr 1;8:609217. doi: 10.3389/fnut.2021.609217 (PMC8047079; doi:10.3389/fnut.2021.609217)

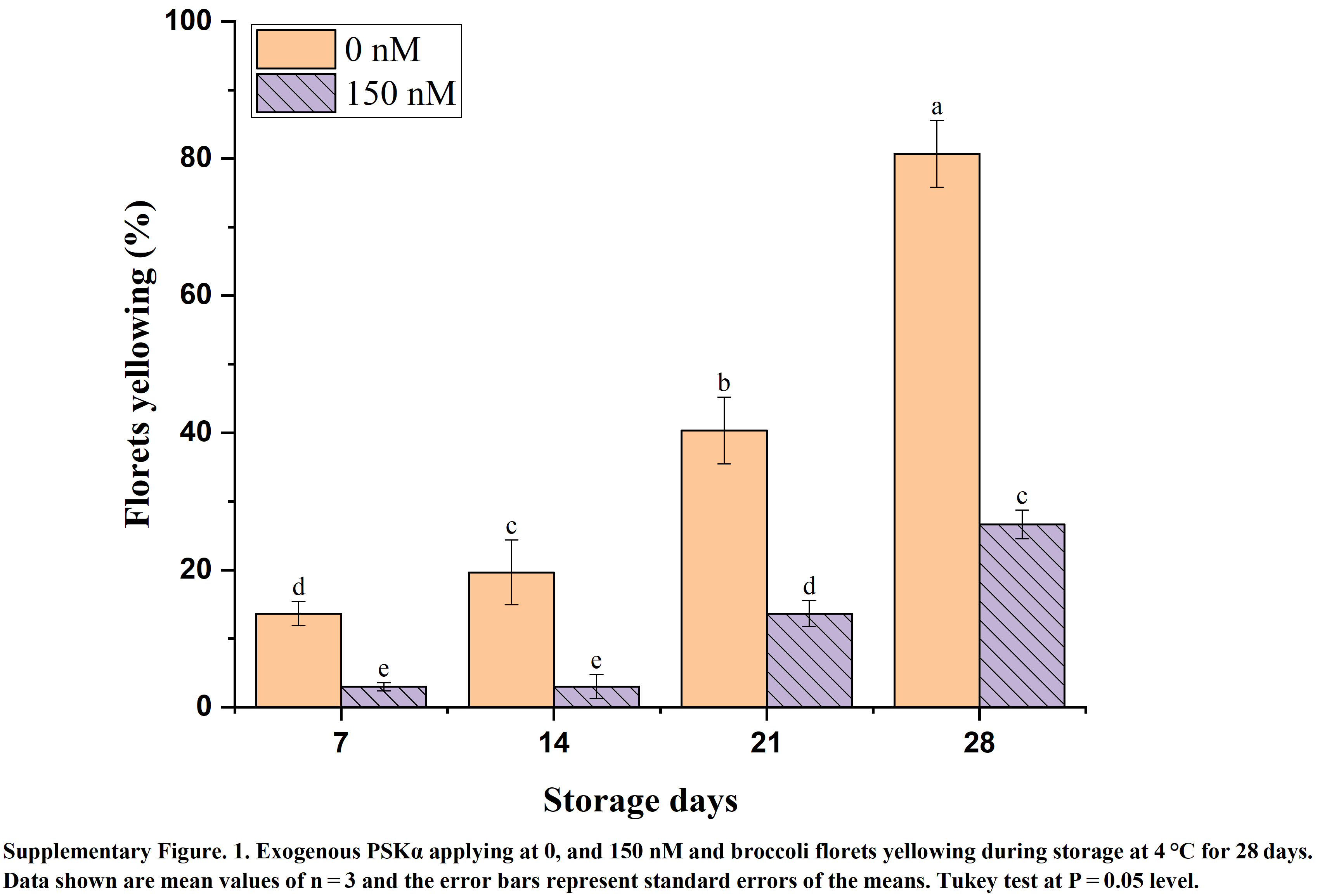

Supplement: Supplementary file 1 [file Image_1.jpg]
