# Supplementary material for: Delaying Broccoli Floret Yellowing by Phytosulfokine α Application During Cold Storage
Source: Front Nutr. 2021 Apr 1;8:609217. doi: 10.3389/fnut.2021.609217 (PMC8047079; doi:10.3389/fnut.2021.609217)
Supplement: Supplementary file 2 [file Table_1.doc]

**Supplementary Table. 1.** The primers used for genes expression by qRT-PCR.

| **Genes** | **Accession numbers** | **Functional annotations** | **Primer sequences (5′-3′)** | **Amplicon size (bp)** |
| --- | --- | --- | --- | --- |
| *ACS1* | X82273 | Ethylene biosynthesis | F: GGTTCCGACTCCTTATTATCCAG  R: TACCGAGCGGGTTGGAAGG | 200 |
| *ACO1* | X81628 | Ethylene biosynthesis | F: GCTTGCCTGTAAAGAAACCG  R: TACGGCTGCTGTTGGGTT | 151 |
| *PPH* | HQ840431 | Chlorophyll degradation | F: AGAGGTTATCGGTGAGCCA  R: GACGAGATGAGGATGGG | 91 |
| *PAO* | AM388844 | Chlorophyll degradation | F: GCGAAATTCCCGTCCAGAGTCTC  R: TTATCTCCGCCGTGCTCTTCTTC | 143 |
| *LCD* | NC027753 | H_2_S biosynthesis | F: GGAGGACACTGTGGTGATGT  R: CGTTCGCTCTACCTTTCTTT | 176 |
| *DCD* | NC027754 | H_2_S biosynthesis | F: AGAAGAGTCGTGTCAGGTTTG  R: CAGGGAAGCCCTAAAGACATAG | 91 |
| *Actin* | AF044573 | Housekeeping gene | F: CCAGAGGTCTTGTTCCAGCCATC  R: GTTCCACCACTGAGCACAATGTTAC | 137 |
